# Annotations

**Published:** 1897-01-30

**Authors:** 


### Annotations.

### Grubbing and Tippling.

In an article in the *New Review* on the Public School Product, the head master of Loretto, H. H. Almond, LL.D., makes a suggestion which we think is of some importance, to the effect that the frequent recourse to the "tuck shop," so common at some public schools, leads in later life to an equally frequent recourse to stimulants. The ruination of digestion which results from the intermediate meals which boys so love is a thing to be deplored, and its effect upon the health and physique are, as is well known, made all the worse by the fact that those who get into the habit of trusting to the stuff they buy are only too ready to neglect the healthier food supplied to them at the proper times. There can, however, be little doubt that the habit of constantly stimulating the mucous membrane by tasty morsels at all hours of the day tends also to produce a sense of want and craving whenever the stomach is not actually at work on something or another. It is far easier to develop a morbid action of that organ, and a morbid irritability of its nervous supply, than it is to cast it off. The days of tuck shops pass, but the habit of wanting frequent comfort for the stomach remains, especially where the mode of life is sedentary. It is not, however, satisfied so innocently; the tart and the bun give place to the "peg," the "nip," the liqueur, or whatever form of alcohol emancipated youth chooses to burn its stomach with. We agree with this wise head master in advising that however much the products of the tuck shop may be introduced at meal times, especially in the form of fruit, which he would by no means prohibit, an absolute veto should be put upon the evil habit of boys feeding between meals.

### Physiology and Cold Feet.

The difference between cold feet and warm is a difference which has an important effect upon the general temperature of the body, and an equally important effect upon both health and comfort, alike in the night and in the day. The blood, propelled by the heart, circulates, as we know, throughout the whole body. If a considerable portion of the body, like the two feet, is very cold, the warm blood has to pass through a large cold area, and it becomes cooled in passing. But in the case of cold feet, not only is the area cold, but the flow of blood, in consequence of the remoteness of the feet from the cardiac pump, is at its lowest. There is, consequently, the element of time to be considered; and, when this is considered, it is perceived how very much really cold feet may lower the whole temperature of the blood, and so of the body. On the other hand, when the feet are warm, very warm, they will not lower, but actually raise, the temperature of the blood, and thus make a very important difference indeed in the warmth of the whole body. Thinking of these things, one may readily see why a small degree of coldness in the feet may produce in some persons colic pains, and even diarrhœa, whilst a greater degree of coldness in the feet may bring about in others even a typhlitis or peritonitis. Now, it is not worth while for any of us in the present wintry season to run the risks of serious discomfort and of grave illness if we can prevent such risks. As a matter of fact we can, and ought to do, both by day and by night. In these columns we have often advised the aged and those of feeble circulation to resort to a hot bottle or to wrap their feet in flannel during the hours of sleep. Our more immediate purpose at the present time is to suggest the necessity for and a means of keeping the feet warm during the day for persons who have to stand in the street, sit in ill-warmed offices, or travel by train, and so on. Snow-shoes, indiarubber snow-shoes, lined with skin, or even flannel, furnish a sovereign remedy for cold feet. The writer has been surprised at the boldness with which certain aged people, gouty men, and delicate women of his acquaintance have faced the recent snowy and slushy roads when armed, or rather "footed," with snow-shoes. The means proposed are simple, and as scientific as simple; but the comfort and safety secured by their use cannot be valued in money.

### Science and the Fragrant Weed.

The muttered grumblings of the smokers have long been heard in regard to those rigid rules of strict propriety by which the meetings of the various medical societies are governed. Not altogether without some show of reason do they ask why smoking should be prohibited. They say that when they want to think or write at home they always smoke; that while driving from patient to patient they take a cigarette, and deal with their patient all the better for it; and that when the work of the day is over they must have their pipe. Now these meetings are all post prandial affairs; and it is whispered that in the struggle between the after-dinner smoke and the after-dinner science, it is the science which goes to the wall. It is even suggested that some of the dulness, which even the dullest of us must observe to be predominant at some of our meetings, is due to the fact that many of the brightest spirits in the world of medicine are also votaries of nicotine, and that in her favour they give up the sterner cult. To remedy this state of affairs it is proposed by the Council of the Harveian Society that smoking shall be permitted at all ordinary meetings except those at which the presence of patients is required. The fateful decision on this important question will be taken at the next meeting on February 4th, when, perhaps, we may see the thin end of the wedge introduced which shall lead to less formality and more usefulness in the meetings of other societies; for, be it observed the Harveian is far from being the dullest of them, the papers being often of great excellence, and the secretaries apparently taking some trouble to ensure that a proper discussion shall take place. It rarely happens at the Harveian that a paper fizzles out without a word of comment, as is too often the case at other centres of learning which we need not mention. To us it seems that the whole question ought to resolve itself into a matter of ventilation! We would be the last to suggest that a scientific Codgers Hall should be established in Hanover Square; but if it can be so arranged that smoking can be indulged in without offence to those who love not the weed, we can clearly see that many would attend the meetings who at present decline to forego the pipe. Seeing that the greatest of the societies already allows the wild dissipation of tea in the library, is it too much to look forward to seeing smoking permitted in the lecture hall?

